# Magnonic Floquet Quantum Spin Hall Insulator in Bilayer Collinear Antiferromagnets

**DOI:** 10.1038/s41598-019-43702-9

**Published:** 2019-05-10

**Authors:** S. A. Owerre

**Affiliations:** Perimeter Institute for Theoretical Physics, 31 Caroline St. N., Waterloo, Ontario N2L 2Y5 Canada

**Keywords:** Magnetic properties and materials, Topological insulators

## Abstract

We study irradiated two-dimensional insulating bilayer honeycomb ferromagnets and antiferromagnets coupled antiferromagnetically with a zero net magnetization. The former is realized in the recently synthesized bilayer honeycomb chromium triiodide CrI_3_. In both systems, we show that circularly-polarized electric field breaks time-reversal symmetry and induces a dynamical Dzyaloshinskii-Moriya interaction in each honeycomb layer. However, the resulting bilayer antiferromagnetic system still preserves a combination of time-reversal and space-inversion (${\bf{P}}{\bf{T}}$) symmetry. We show that the magnon topology of the bilayer antiferromagnetic system is characterized by a ${{\bf{Z}}}_{{\bf{2}}}$ Floquet topological invariant. Therefore, the system realizes a magnonic Floquet quantum spin Hall insulator with spin filtered magnon edge states. This leads to a non-vanishing Floquet magnon spin Nernst effect, whereas the Floquet magnon thermal Hall effect vanishes due to ${\bf{P}}{\bf{T}}$ symmetry. We study the rich ${{\bf{Z}}}_{{\bf{2}}}$ Floquet topological magnon phase diagram of the system as a function of the light amplitudes and polarizations. We further discuss the great impact of the results on future experimental realizations.

## Introduction

In recent years, periodically driven solid-state materials have emerged as an alternative avenue to extend the search for topological quantum materials^1–30^. This mechanism involves the exposure of a topologically trivial quantum material to a time-periodic electric field. In this system, time-reversal symmetry of the Bloch bands is broken by circularly-polarized electric field by modifying the intrinsic properties of the material via light-matter interactions. This results in a Floquet Chern insulator such as in irradiated graphene^1,5^. The non-equilibrium topological systems are believed to give interesting properties that are not possible in the equilibrium systems.

In insulating magnets, the quantum theory of magnons dictates that magnons carry a spin magnetic dipole moment and an intrinsic spin of 1, which can be used for dissipationless information processing in the emerging field of magnon spintronics^31,32^. This implies that magnons can accumulate the Aharonov-Casher phase^33–38^ when exposed to a time-independent spatially-varying electric field resulting in magnonic Landau levels^38^ and chiral anomaly in Weyl magnons^39,40^. Remarkably, the magnon accumulated Aharonov-Casher phase has a strikingly different physics when the electric field is time-dependent and periodic as in electronic systems. In this case, the resulting irradiated insulating magnets can be investigated using the Floquet theory in a similar manner to irradiated metallic electronic systems. Unlike electronic systems, the magnetic Floquet physics can reshape the underlying spin Hamiltonian to stabilize magnetic phases and provides a promising avenue for inducing and tuning Floquet topological spin excitations^41–43^, with a direct implication of generating and manipulating ultrafast spin current using terahertz (THz) radiation^44–46^. In this respect, the concept of magnonic Floquet Chern insulator has emerged^41–43^, where circularly-polarized light induces a dynamical Dzyaloshinskii-Moriya (DM) interaction^47–49^ in a single-layer two-dimensional (2D) insulating honeycomb ferromagnet. This approach has also been generalized to engineer Floquet Weyl magnons^50^ in three-dimensional (3D) insulating honeycomb ferromagnets. Similar to electronic Floquet system, time-reversal symmetry is broken by circularly-polarized electric field and the Floquet topological system is characterized by the first Chern number. Therefore, the topological aspects of electronic and magnonic Floquet systems are essentially the same and they originate from the same oscillating time-periodic electric field. To make this similarity obvious, we note that in the magnonic Floquet topological systems, the intensity of light is characterized by the dimensionless quantity^41^

1 $${ {\mathcal E} }_{i}=\frac{g{\mu }_{B}{E}_{i}a}{\hslash {c}^{2}},$$

where *E*_*i*_ ($i=x,y$) are the amplitudes of the electric field, *g* is the Landé g-factor, *μ*_*B*_ is the Bohr magneton, *a* is the lattice constant, *ħ* and *c* are the reduced Plank’s constant and the speed of light respectively. The dimensionless quantity in Eq. (1) should be compared to that of electronic Floquet topological systems^1,2,5^

2 $${ {\mathcal E} }_{i}=\frac{e{E}_{i}a}{\hslash \omega },$$

where *e* is the electron charge and $\omega$ is the angular frequency of light. Thus, for the irradiated magnetic insulators we can identify the spin magnetic dipole moment carried by magnon as

3 $$g{\mu }_{B}=\frac{e{c}^{2}}{\omega }=\frac{ec\lambda }{2\pi }.$$

Therefore, we can see that for a typical light wavelength *λ* of order 10^−8^m, the spin magnetic dipole moment *gμ*_*B*_ carried by magnon in the irradiated magnetic insulators is comparable to the electron charge *e*. This shows the similarity between the electronic and the magnonic Floquet topological systems.

Recently, the ${{\mathbb{Z}}}_{2}$ characterization of topological magnon bands in the equilibrium time-independent insulating antiferromagnets has garnered considerable attention^51–56^. In particular, for the 2D insulating bilayer honeycomb antiferromagnets with a DM interaction^47–49^ the system preserves time-reversal and space-inversion (${\mathscr{P}}{\mathscr{T}}$) symmetry and realizes the magnonic analog of ${{\mathbb{Z}}}_{2}$ topological insulator^57–59^. Unfortunately, most 2D insulating honeycomb antiferromagnets do not have the unique form of the required DM interaction^47^. In fact, the absence of this unique DM interaction in most insulating honeycomb antiferromagnets has prevented a discernible experimental observation of the magnon spin Nernst voltage in MnPS_3_^60^. One possible mechanism to induce the unique form of the required DM interaction in 2D insulating honeycomb antiferromagnets is through photo-irradiation with a circularly-polarized electric field^41^.

In this report, we propose a ${{\mathbb{Z}}}_{2}$ magnonic Floquet quantum spin Hall insulator in bilayer collinear antiferromagnets with ${\mathscr{P}}{\mathscr{T}}$ symmetry. Specifically, we study irradiated 2D insulating bilayer honeycomb ferromagnets and antiferromagnets coupled antiferromagnetically with a zero net magnetization, where the former is realized in bilayer CrI_3_^61–63^. Our theoretical formalism is based on the Floquet theory, spin-wave theory, and quantum field theory. In both honeycomb bilayer systems, we show that circularly-polarized electric field induces a dynamical DM interaction in each honeycomb layer, but the bilayer antiferromagnetic system preserves ${\mathscr{P}}{\mathscr{T}}$ symmetry, hence the resulting magnon topology is characterized by a ${{\mathbb{Z}}}_{2}$ Floquet topological invariant quantity. We obtain the ${{\mathbb{Z}}}_{2}$ Floquet topological magnon phase diagram and identify the regimes where the Floquet magnon spin Nernst coefficient changes sign. We also show that both systems exhibit Floquet spin-filtered magnon edge states, where Floquet magnon with opposite spin propagates in opposite directions. Our results provide a powerful mechanism for manipulating the intrinsic properties of 2D insulating honeycomb antiferromagnetic materials such as bilayer CrI_3_, and could pave the way for studying new interesting features in 2D insulating antiferromagnets such as photo-magnonics^64^, magnon spintronics^31,32^, and ultrafast optical control of magnetic spin currents^37,44–46^.

## Results

### Bilayer Heisenberg spin model

**Figure 1:**
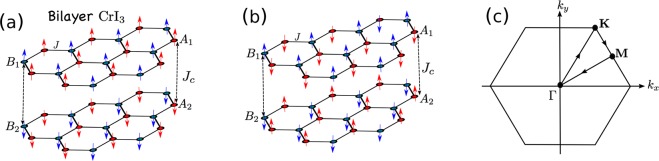
(**a**) Case (*i*) – bilayer honeycomb-lattice ferromagnets coupled antiferromagnetically as realized in bilayer CrI_3_. (**b**) Case (*ii*) – bilayer honeycomb-lattice antiferromagnets coupled antiferromagnetically. (**c**) The Brillouin zone (BZ) of the honeycomb lattice with high-symmetry paths.

We consider the Heisenberg spin model for 2D insulating bilayer honeycomb ferromagnets and antiferromagnets coupled antiferromagnetically. The Hamiltonian is governed by

4 $${\mathscr{H}}=J\,\sum _{\langle ij\rangle ,\ell }\,{\vec{S}}_{i,\ell }\cdot {\vec{S}}_{j,\ell }+{J}_{c}\,\sum _{i}\,{\vec{S}}_{i}^{T}\cdot {\vec{S}}_{i}^{B},$$

where ${\vec{S}}_{i}$ is the spin vector at site *i* and $\ell$ labels the top (*T*) and bottom (*B*) layers. We will consider two different cases: (*i*) $J < 0$, ${J}_{c} > 0$ (see Fig. 1(a)). (*ii*) $J > 0$, ${J}_{c} > 0$ (see Fig. 1(b)). The intralyer coupling is ferromagnetic in case (*i*) and antiferromagnetic in case (*ii*). In both cases the net magnetization vanishes. We note that case (*i*) is manifested in the bilayer honeycomb magnet CrI_3_^61–63^. There are four sublattices in the unit cell denoted by *A*_1_, *B*_1_, *A*_2_, *B*_2_. In both cases the interlayer exchange couples sites on the sublattices ${A}_{1}\leftrightarrow {A}_{2}$ and ${B}_{1}\leftrightarrow {B}_{2}$.

### Bosonic Bogoliubov-de Gennes model

We will focus on the low-temperature regime, when the magnetic excitations of the spin Hamiltonian in Eq. (4) can be described by the Holstein Primakoff transformation^65^ (see Methods). The bosonic Hamiltonian in momentum space is given by

5 $${\mathscr{H}}=\frac{1}{2}\,\sum _{\vec{k}}\,({u}^{\dagger }(\vec{k}),u(-\vec{k}))\cdot {\mathscr{H}}(\vec{k})\cdot (\begin{array}{c}u(\vec{k})\\ {u}^{\dagger }(-\vec{k})\end{array}).$$

The bosonic Bogoliubov-de Gennes (BdG) Hamiltonian is given by

6 $${\mathscr{H}}(\vec{k})=(\begin{array}{cc}{{\mathscr{H}}}_{+}(\vec{k}) & 0\\ 0 & {{\mathscr{H}}}_{-}(\vec{k})\end{array}),$$

with ${{\mathscr{H}}}_{-}(\vec{k})={{\mathscr{H}}}_{+}^{\ast }(-\vec{k})$. Each block Hamiltonian is a 4 × 4 Hermitian matrix representing the ${S}_{z}=+\,1$ and the ${S}_{z}=-\,1$ spin sectors. Therefore, ${\mathcal H} (\vec{k})$ is invariant under ${\mathscr{P}}{\mathscr{T}}$ symmetry given by ${\mathscr{P}}{\mathscr{T}}={\sigma }_{x}\otimes {\sigma }_{0}{\mathscr{K}}$, where ${\mathscr{K}}$ is complex conjugation, *σ*_*i*_ are Pauli matrices with an identity *σ*_0_. The ${S}_{z}=+\,1$ sector Hamiltonian in case (*i*) ($J < 0$, ${J}_{c} > 0$) is given by

7 $${{\mathscr{H}}}_{+}^{(i)}(\vec{k})=(\begin{array}{cccc}|{v}_{0}| & -\,|{v}_{J}|\,{f}^{\ast }(\vec{k}) & {v}_{{J}_{c}} & 0\\ -\,|{v}_{J}|\,f(\vec{k}) & |{v}_{0}| & 0 & {v}_{{J}_{c}}\\ {v}_{{J}_{c}} & 0 & |{v}_{0}| & -\,|{v}_{J}|\,f(\vec{k})\\ 0 & {v}_{{J}_{c}} & -\,|{v}_{J}|\,{f}^{\ast }(\vec{k}) & |{v}_{0}|\end{array}),$$

with ${u}^{\dagger }(\vec{k})=({a}_{\vec{k}{A}_{1}}^{\dagger },{a}_{\vec{k}B1}^{\dagger },{a}_{-\vec{k}{A}_{2}},{a}_{-\vec{k}B2})$. The ${S}_{z}=+\,1$ sector Hamiltonian in case (*ii*) ($J > 0$, ${J}_{c} > 0$) is given by

8 $${{\mathscr{H}}}_{+}^{(ii)}(\vec{k})=(\begin{array}{cccc}{v}_{0} & 0 & {v}_{J}\,{f}^{\ast }(\vec{k}) & {v}_{{J}_{c}}\\ 0 & {v}_{0} & {v}_{{J}_{c}} & {v}_{J}\,f(\vec{k})\\ {v}_{J}\,f(\vec{k}) & {v}_{{J}_{c}} & {v}_{0} & 0\\ {v}_{{J}_{c}} & {v}_{J}\,{f}^{\ast }(\vec{k}) & 0 & {v}_{0}\end{array}),$$

with $${u}^{\dagger }(\vec{k})=({a}_{\overrightarrow{k}{A}_{1}}^{\dagger },{a}_{\overrightarrow{k}B2}^{\dagger },{a}_{-\overrightarrow{k}{B}_{1}},{a}_{-\overrightarrow{k}A2})$$.

**Figure 2:**
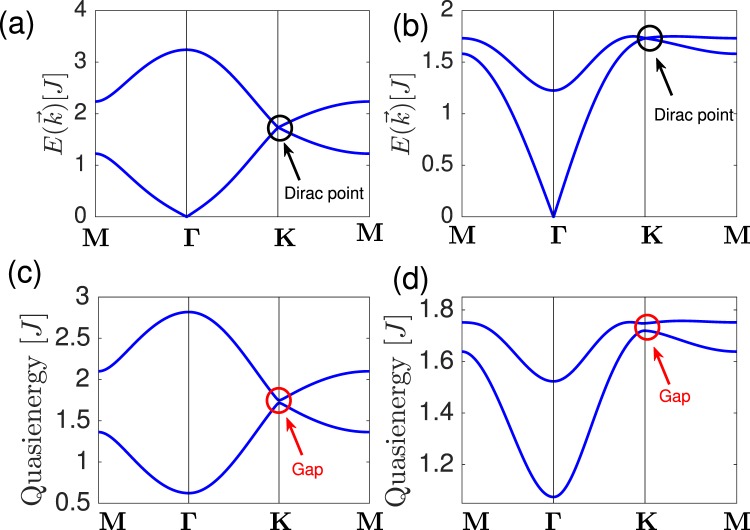
Top panel. Equilibrium doubly-degenerate antiferromagnetic Dirac magnon bands for (**a**) case (*i*) and (**b**) case (*ii*) with ${J}_{c}$/$J=0.5$ and $S=1$/2. The Dirac points are indicated by circles. Bottom panel. Floquet doubly-degenerate topological magnon bands for (**c**) case (*i*) and (**d**) case (*ii*). The parameters are ${J}_{c}$/$J=0.5$, $S=1$/2, ${ {\mathcal E} }_{x}={ {\mathcal E} }_{y}=1$, $\varphi =\pi$/2, and $\hslash \omega$/$J=10$. The red circle points indicate the massive Floquet Dirac magnon.

Here ${v}_{0}=3{v}_{J}+{v}_{{J}_{c}}$, ${v}_{J}=JS$, ${v}_{{J}_{c}}={J}_{c}S$ and $f(\vec{k})={\sum }_{\ell }\,{e}^{i{k}_{\ell }}$ with ${k}_{\ell }=\vec{k}\cdot {\vec{a}}_{\ell }$. The primitive lattice vectors are ${\vec{a}}_{1}=a\sqrt{3}\hat{x}$, ${\vec{a}}_{2}=a(\sqrt{3}\hat{x}/2+3\hat{y}/2)$, ${\vec{a}}_{3}=0$. The nearest-neighbour vectors are ${\vec{\delta }}_{1,2}=a(\mp \sqrt{3}\hat{x}/2+\hat{y}/2)$, ${\vec{\delta }}_{3}=-\,a\hat{y}$. The Hamiltonian can be diagonalized by paraunitary operators. The magnon bands are doubly-degenerate due to ${\mathscr{P}}{\mathscr{T}}$ symmetry and they are given by

9 $${E}_{\sigma \eta }^{(i)}(\vec{k})=\sqrt{{(3{v}_{J}+|{v}_{{J}_{c}}|+\eta {v}_{J}|f(\vec{k})|)}^{2}-|{v}_{{J}_{c}}{|}^{2}},$$

10 $${E}_{\sigma \eta }^{(ii)}(\vec{k})=\sqrt{{(3{v}_{J}+{v}_{{J}_{c}})}^{2}-{({v}_{{J}_{c}}+\eta {v}_{J}|f(\vec{k})|)}^{2}},$$

where $\sigma =\pm$ for the layers and $\eta =\pm$ for the sublattices. The magnon energy bands are depicted in Fig. 2(a and b) respectively. In both cases the linear Goldstone mode at the ${\boldsymbol{\Gamma }}$-point signifies antiferromagnetic order. The doubly-degenerate antiferromagnetic Dirac magnons occur at the **K**-point in both cases.

### Irradiated bilayer antiferromagnetic insulator

We will now present the analysis of irradiated 2D insulating antiferromagnets. Let us consider the effects of an oscillating electric field $\vec{E}(\tau )$, irradiated perpendicular to the 2D insulating antiferromagnets. The consequence of irradiated insulating antiferromagnets is that hopping magnon with spin magnetic dipole moment *gμ*_*B*_$\hat{z}$ will accumulate the time-dependent version of the Aharanov-Casher phase^33^ given by (see Methods)

11 $${{\rm{\Phi }}}_{ij,\ell }^{s}(\tau )=\hat{s}{\mu }_{m}\,{\int }_{{\vec{r}}_{i}}^{{\vec{r}}_{j}}\,\vec{{\rm{\Xi }}}(\tau )\cdot d\vec{\ell },$$

where $\hat{s}={\rm{diag}}({\rm{I}},-\,{\rm{I}})$, ${\mu }_{m}=g{\mu }_{B}$/$\hslash {c}^{2}$, and ${\vec{r}}_{i}$ is the spin position at site *i*. We have used the notation $\vec{{\rm{\Xi }}}(\tau )=\vec{E}(\tau )\times \hat{z}$ for brevity and $\vec{E}(\tau )=-{{\rm{\partial }}}_{\tau }\vec{A}(\tau )$, where $\vec{A}(\tau )$ is the time-dependent vector potential given by

12 $$\vec{A}(\tau )=[-{A}_{x}\,\cos (\omega \tau +\varphi ),{A}_{y}\,\cos (\omega \tau ),0],$$

where ${A}_{i}={E}_{i}$/$\omega$ ($i=x,y$) are the strength of the time-dependent vector potential and $\varphi$ is the phase difference. For circularly-polarized electric field $\varphi =\pi$/2 and for linearly-polarized electric field $\varphi =0$ or *π*. The corresponding time-dependent oscillating electric field is given by

13 $$\vec{{\rm{\Xi }}}(\tau )=[{E}_{y}\,\sin (\omega \tau ),{E}_{x}\,\sin (\omega \tau +\varphi ),0].$$

The resulting time-dependent Hamiltonian is given by

14 $${\mathscr{H}}(\tau )=J\,\sum _{\langle ij\rangle ,\ell }\,[{S}_{i,\ell }^{z}{S}_{j,\ell }^{z}+\frac{1}{2}({S}_{i,\ell }^{+}{S}_{j,\ell }^{-}{e}^{i{{\rm{\Phi }}}_{ij,\ell }^{s}(\tau )}+{\rm{H}}.{\rm{c}}.)]+{J}_{c}\,\sum _{i}\,{\overrightarrow{S}}_{i}^{T}\cdot {\overrightarrow{S}}_{i}^{B},$$

where ${S}_{i}^{\pm (\ell )}={S}_{i,\ell }^{x}\pm i{S}_{i,\ell }^{y}$ denote the spin raising and lowering operators. Note that the interlayer coupling is not affected by light intensity. The spin current can be derived as

15 $${J}^{S}=\frac{\partial  {\mathcal H} (\tau )}{\partial {{\rm{\Phi }}}_{ij,\ell }(\tau )}\equiv \sum _{j\in i;\ell }\,{j}_{ij,\ell }^{s},$$

where ${j}_{ij,\ell }^{s}=-\,i\frac{J}{2}{e}^{i{{\rm{\Phi }}}_{ij,\ell }(\tau )}{S}_{i,\ell }^{-}{S}_{j,\ell }^{+}+{\rm{H}}.{\rm{c}}.$ Thus, the time-dependent Aharonov-Casher phase ${{\rm{\Phi }}}_{ij}(\tau )$ acts as a vector potential or gauge field to the spin current.

The Floquet theory is a powerful mechanism to study periodically driven quantum systems^1–30^. It enables one to transform a time-dependent periodic Hamiltonian into a static effective Hamiltonian governed by the Floquet Hamiltonian. In the off-resonant limit, when the photon energy *ħ*$\omega$ is greater than the energy scale of the static system, the effective static Hamiltonian is given by^5–7^

16 $${ {\mathcal H} }_{eff}\approx { {\mathcal H} }_{0}+{\rm{\Delta }}{ {\mathcal H} }_{eff},$$

where ${\rm{\Delta }}{ {\mathcal H} }_{eff}=[{ {\mathcal H} }_{1},{ {\mathcal H} }_{-1}]$/$\hslash \omega$ is the photon emission and absorption term. We use the discrete Fourier component of the time-dependent Hamiltonian ${ {\mathcal H} }_{n}=\frac{1}{T}\,{\int }_{0}^{T}\,d\tau {e}^{-in\omega \tau } {\mathcal H} (\tau )$ with period $T=2\pi$/$\omega$. For circularly-polarized light $\varphi =\pi$/2 and ${E}_{x}={E}_{y}={E}_{0}$, we obtain

17 $$\begin{array}{ccc}{{\mathscr{H}}}_{n} & = & J\,\sum _{\langle ij\rangle ,\ell }\,[\frac{{\mathscr{J}}({{\mathscr{E}}}_{0})}{2}({S}_{i,\ell }^{-}{S}_{j,\ell }^{+}{e}^{-in{\theta }_{ij,\ell }}+H.c.)+{\delta }_{n,0}{S}_{i,\ell }^{z}{S}_{j,\ell }^{z}]\\  &  & +\,{J}_{c}{\delta }_{n,0}\,\sum _{i}\,{\vec{S}}_{i}^{T}\cdot {\vec{S}}_{i}^{B},\end{array}$$

where ${ {\mathcal E} }_{0}=g{\mu }_{B}{E}_{0}a$/$\hslash {c}^{2}$ is the dimensionless Floquet parameter, ${\theta }_{ij,\ell }$ is the relative angle between ${\vec{r}}_{i}$ and ${\vec{r}}_{j}$, ${{\mathscr{J}}}_{n}(x)$ is the Bessel function of order $n\in {\mathbb{Z}}$, and ${\delta }_{n,\ell }=1$ for $n=\ell$ and zero otherwise. The zeroth-order term is given by

18 $${{\mathscr{H}}}_{0}=\sum _{\langle ij\rangle ,\ell }\,[J{{\mathscr{J}}}_{0}({{\mathscr{E}}}_{0})\,({S}_{i,\ell }^{x}{S}_{j,\ell }^{x}+{S}_{i,\ell }^{y}{S}_{j,\ell }^{y})+J{S}_{i,\ell }^{z}{S}_{j,\ell }^{z}]+{J}_{c}\,\sum _{i}\,{\vec{S}}_{i}^{T}\cdot {\vec{S}}_{i}^{B},$$

which is an *XXZ* Heisenberg spin model, where $J{{\mathscr{J}}}_{0}({ {\mathcal E} }_{0}) < J$ for ${ {\mathcal E} }_{0}\ne 0$. The first-order term Δ${ {\mathcal H} }_{eff}$ involves the commutation relation $[{S}_{\alpha }^{+}{S}_{\beta }^{-},{S}_{\rho }^{+}{S}_{\gamma }^{-}]$ = $2({\delta }_{\beta \rho }{S}_{\beta }^{z}{S}_{\alpha }^{+}{S}_{\gamma }^{-}-{\delta }_{\alpha \gamma }{S}_{\alpha }^{z}{S}_{\rho }^{+}{S}_{\beta }^{-})$, which gives rise to a photoinduced DM interaction^41^ of the form

19 $${\rm{\Delta }}{{\mathscr{H}}}_{eff}={D}_{F}\,\sum _{\langle \langle ijk\rangle \rangle ,\ell }\,{\nu }_{jk}{\vec{S}}_{i,\ell }\cdot ({\vec{S}}_{j,\ell }\times {\vec{S}}_{k,\ell }),$$

where ${\vec{S}}_{i,\ell }={S}_{i,\ell }^{z}\hat{z}$, ${D}_{F}=\sqrt{3}{[J{{\mathscr{J}}}_{1}({ {\mathcal E} }_{0})]}^{2}$/$\hslash \omega$, and ${\nu }_{jk}=\pm \,1$ for the two triangular plaquettes on the next-nearest neighbour bonds of the honeycomb lattice. Therefore, time-reversal symmetry of each honeycomb layer is broken by circularly-polarized light through a photoinduced DM interaction, but the bilayer antiferromagnetic system still preserves ${\mathscr{P}}{\mathscr{T}}$ symmetry. Thus, magnonic Floquet quantum spin Hall insulator can arise in irradiated bilayer collinear antiferromagnets. On the contrary, linearly-polarized light does not break time-reversal symmetry, thus Δ${ {\mathcal H} }_{eff}=0$ for $\varphi =0$.

### Periodically-driven bosonic BdG model

In this section, we will study the magnon band structures for a general light polarization $\varphi \in [0,2\pi ]$ and a general amplitude ${E}_{x}\ne {E}_{y}$. It is advantageous to periodically drive the bosonic BdG Hamiltonian in Eqs (7) and (8). In this case, the Aharanov-Casher phase enters the momentum space Hamiltonian through the time-dependent Peierls substitution $\vec{k}\to \vec{k}-\hat{s}{\mu }_{m}\vec{{\rm{\Xi }}}(\tau )$. Using the analysis outline in Methods, we have obtained the Fourier decomposition of the single particle bosonic BdG Hamiltonian, which enters the time-dependent Schrödinger equation. For the ${S}_{z}=+\,1$ sector, the Fourier Hamiltonian for case (*i*) (i.e. $J < 0$, ${J}_{c} > 0$) is given by

20 $${{\mathscr{H}}}_{+,q}^{(i)}(\vec{k})=(\begin{array}{cccc}|{v}_{0}|{\delta }_{q,0} & -{\rho }_{-q}^{\ast }(\vec{k}) & {v}_{{J}_{c}}{\delta }_{q,0} & 0\\ -{\rho }_{q}(\vec{k}) & |{v}_{0}|{\delta }_{q,0} & 0 & {v}_{{J}_{c}}{\delta }_{q,0}\\ {v}_{{J}_{c}}{\delta }_{q,0} & 0 & |{v}_{0}|{\delta }_{q,0} & -{\rho }_{q}(\vec{k})\\ 0 & {v}_{{J}_{c}}{\delta }_{q,0} & -{\rho }_{-q}^{\ast }(\vec{k}) & |{v}_{0}|{\delta }_{q,0}\end{array}),$$

and the Fourier Hamiltonian for case (*ii*) (*i*.*e*. $J > 0$, ${J}_{c} > 0$) is given by

21 $${{\mathscr{H}}}_{+,q}^{(ii)}(\vec{k})=(\begin{array}{cccc}{v}_{0}{\delta }_{q,0} & 0 & {\rho }_{-q}^{\ast }(\vec{k}) & {v}_{{J}_{c}}{\delta }_{q,0}\\ 0 & {v}_{0}{\delta }_{q,0} & {v}_{{J}_{c}}{\delta }_{q,0} & {\rho }_{q}(\vec{k})\\ {\rho }_{q}(\vec{k}) & {v}_{{J}_{c}}{\delta }_{q,0} & {v}_{0}{\delta }_{q,0} & 0\\ {v}_{{J}_{c}}{\delta }_{q,0} & {\rho }_{-q}^{\ast }(\vec{k}) & 0 & {v}_{0}{\delta }_{q,0}\end{array}),$$

where $q\in {\mathbb{Z}}$ and ${\rho }_{q}(\vec{k})={\sum }_{\ell }\,{t}_{\ell ,q}{e}^{i{k}_{\ell }}$. The renormalized Heisenberg exchange interactions are given by

22 $$\begin{array}{rcl}{t}_{1,q} & = & |{v}_{J}|{{\mathscr{J}}}_{-q}({ {\mathcal E} }_{-}){e}^{-iq{{\rm{\Psi }}}_{-}},\\ {t}_{2,q} & = & |{v}_{J}|{{\mathscr{J}}}_{q}({ {\mathcal E} }_{+}){e}^{iq{{\rm{\Psi }}}_{+}},\\ {t}_{3,q} & = & |{v}_{J}|{{\mathscr{J}}}_{q}({ {\mathcal E} }_{x}){e}^{iq\varphi }.\end{array}$$

23 $${ {\mathcal E} }_{\pm }=\frac{1}{2}\sqrt{3{ {\mathcal E} }_{y}^{2}+{ {\mathcal E} }_{x}^{2}\pm 2\sqrt{3}{ {\mathcal E} }_{x}{ {\mathcal E} }_{y}\,\cos (\varphi )}\,{\rm{and}}\,{{\rm{\Psi }}}_{\pm }=\arctan (\frac{{ {\mathcal E} }_{x}\,\sin (\varphi )}{\sqrt{3}{ {\mathcal E} }_{y}\pm { {\mathcal E} }_{x}\,\cos (\varphi )}).$$

In the off-resonant limit $\hslash \omega \gg J$, *J*_*c*_, the system can be described by an effective time-independent Hamiltonian given by^5–7^

24 $${{\mathscr{H}}}_{+}^{eff}(\vec{k})\approx {{\mathscr{H}}}_{+,0}(\vec{k})-\frac{1}{\hslash \omega }[{{\mathscr{H}}}_{+,-1}(\vec{k}),{{\mathscr{H}}}_{+,1}(\vec{k})].$$

The lower block effective Hamiltonian is ${{\mathscr{H}}}_{-}^{eff}(\vec{k})={[{{\mathscr{H}}}_{+}^{eff}(-\vec{k})]}^{\ast }$. The commutator term in Eq. (24) contains terms proportional to $\sin (\vec{k})\,\sin (\varphi )$, which is a mass term to the Dirac magnon. This corresponds to the momentum space of the dynamical DM interaction in Eq. (19) for $\varphi =\pi$/2, and it changes sign in the lower block Hamiltonian ${{\mathscr{H}}}_{-}^{eff}(\vec{k})$.

In Fig. 2(c and d) we have shown the plots of the Floquet magnon quasienergies for $\varphi =\pi$/2 in case (*i*) and case (*ii*) respectively. In both cases, we can see that the Goldstone modes are quadratically gapped out due to SU(2) breaking anisotropy generated by radiation. In addition, the antiferromagnetic Dirac magnon at equilibrium also becomes massive because circularly-polarized electric field induces a dynamical DM interaction in each honeycomb layer, which preserves the ${\mathscr{P}}{\mathscr{T}}$ symmetry of the bilayer antiferromagnetic system as shown in Eq. (19). Therefore, irradiated bilayer antiferromagnetic system is a concrete example of a magnonic Floquet quantum spin Hall insulator, unlike irradiated 2D graphene^1,5^ and 2D insulating ferromagnets^41,42,50^.

### ${{\bf{Z}}}_{{\bf{2}}}$ Topological magnon phase transition

In this section, we will study the topological phase diagram and the topological invariant quantity of the irradiated bilayer antiferromagnetic system. Due to *S*_*z*_ conservation, we can define the block Chern number of the Floquet magnon bands as

25 $${n}_{\sigma }({ {\mathcal E} }_{i},\varphi )=\frac{1}{2\pi }\,\int \,{d}^{2}k\,{{\rm{\Omega }}}_{\sigma }(\overrightarrow{k},{ {\mathcal E} }_{i},\varphi ),$$

where ${{\rm{\Omega }}}_{\sigma }(\vec{k},{{\mathscr{E}}}_{i},\varphi )$ are the Berry curvatures and $\sigma =\pm \,1$ for ${S}_{z}=\pm$ sectors. We can then define the Hall *n*_*H*_ and spin *n*_*S*_ Chern numbers as

26 $${n}_{H}({ {\mathcal E} }_{i},\varphi )={n}_{+}({ {\mathcal E} }_{i},\varphi )+{n}_{-}({ {\mathcal E} }_{i},\varphi )\,{\rm{and}}\,{n}_{S}({ {\mathcal E} }_{i},\varphi )=\frac{1}{2}[{n}_{+}({ {\mathcal E} }_{i},\varphi )-{n}_{-}({ {\mathcal E} }_{i},\varphi )].$$

The ${{\mathbb{Z}}}_{2}$ topological invariant is given by

27 $$\nu ={n}_{S}({ {\mathcal E} }_{i},\varphi )\,{\rm{mod}}\,2.$$

**Figure 3:**
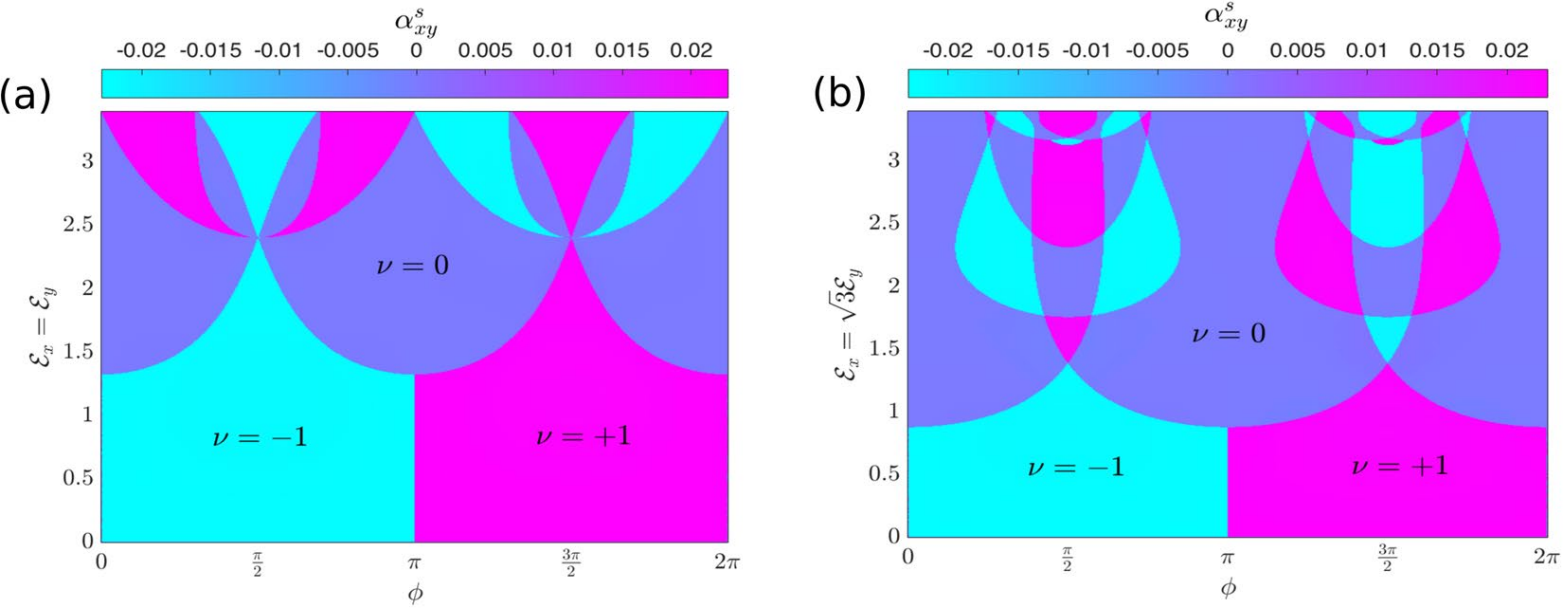
${{\mathbb{Z}}}_{2}$ Floquet topological invariant magnon phase diagram for the lower Floquet magnon band in the irradiated 2D insulating bilayer honeycomb antiferromagnets at high frequency regime $\hslash \omega$/$J=10$ with *J*_*c*_/$J=0.5$. (**a**) ${ {\mathcal E} }_{x}={ {\mathcal E} }_{y}$. (**b**) ${ {\mathcal E} }_{x}=\sqrt{3}{ {\mathcal E} }_{y}$. The colorbar labels the Floquet magnon spin Nernst coefficient at *T*/$J=0.5$. Both panels correspond to case (*ii*).

We have computed the block Chern numbers of the system using the discretized Brillouin zone method^66^. We focus on the lower Floquet quasienergy magnon band. The upper Floquet magnon band can be obtained by flipping the signs. Due to ${\mathscr{P}}{\mathscr{T}}$ symmetry,

28 $${n}_{+}({ {\mathcal E} }_{i},\varphi )=-\,{n}_{-}({ {\mathcal E} }_{i},\varphi ),$$

hence

29 $${n}_{H}({ {\mathcal E} }_{i},\varphi )=0\,{\rm{and}}\,\nu ={n}_{S}({ {\mathcal E} }_{i},\varphi )=-\,1,0,+\,1$$

for both case (*i*) and case (*ii*). The vanishing of ${n}_{H}({ {\mathcal E} }_{i},\varphi )$ implies that the magnon thermal Hall conductivity ${\kappa }_{xy}^{s}$^67–70^ also vanishes in the Floquet bilayer antiferromagnetic system. However, the magnon spin Nernst conductivity^51,71,72^ is nonzero in the single-layer undriven system with DM interaction. Therefore, we will compute the Floquet magnon spin Nernst conductivity for the periodically driven system. We will focus on the regime where the Bose occupation function is close to thermal equilibrium. Hence, we can apply the same formula for the magnon spin Nernst coefficient in undriven system^68–70,72^, which we write as a function of the light parameters ${ {\mathcal E} }_{i}$ and $\varphi$,

30 $${\alpha }_{xy}^{s}({{\mathscr{E}}}_{i},\varphi )=\sum _{\sigma =\pm }\,\int \,\frac{{d}^{2}k}{{(2\pi )}^{2}}\sigma {c}_{1}[g({\epsilon }_{\overrightarrow{k},\alpha }^{\sigma })]\,{{\rm{\Omega }}}_{\sigma }^{\alpha }(\vec{k},{{\mathscr{E}}}_{i},\varphi ),$$

where ${c}_{1}(x)=(1+x)\,\mathrm{ln}(1+x)-x\,\mathrm{ln}\,x$ is the weight function and $g({\epsilon }_{\overrightarrow{k},\alpha }^{\sigma })={[\exp ({\epsilon }_{\overrightarrow{k},\alpha }^{\sigma }/{k}_{B}T)-1]}^{-1}$ is the Bose occupation function close to thermal equilibrium and *α* labels the Floquet magnon bands. Indeed, the Floquet magnon spin Nernst coefficient is simply the ${{\mathbb{Z}}}_{2}$ Floquet topological invariant weighed by the *c*_1_(*x*) function. Note that case (*i*) can be regarded as two copies of the Floquet Chern insulators^41^ with opposite spins, but case (*ii*) is not. Therefore, for case (*ii*) we have shown in Fig. 3 the ${{\mathbb{Z}}}_{2}$ Floquet topological invariant magnon phase diagram of the system for the lower Floquet quasienergy magnon band. A similar phase diagram can be obtained for case (*i*). We can clearly see that in the three regimes where the ${{\mathbb{Z}}}_{2}$ Floquet topological invariant changes sign, the Floquet spin Nernst coefficient also changes sign.

**Figure 4:**
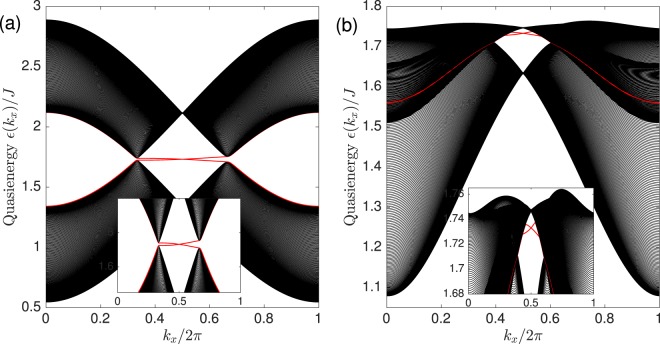
Floquet spin filtered magnon edge states (red lines) for (**a**) case (*i*) and (**b**) case (*ii*). Insets show magnification of the Floquet spin filtered magnon edge states. The parameters are *J*_*c*_/$J=0.5$, $S=1$/2, ${ {\mathcal E} }_{x}={ {\mathcal E} }_{y}=1$ in units of $g{\mu }_{B}a$/$\hslash {c}^{2}$, $\varphi =\pi$/2, and $\hslash \omega$/$J=10$.

To further substantiate the existence of the ${{\mathbb{Z}}}_{2}$ Floquet topological invariant, we show in Fig. 4 the plot of the Floquet magnon bands for a cylindrical strip geometry periodic along the *y* direction and infinite along the *x* direction. We can see that the Floquet magnon edge states traversing the bulk gap are spin filtered. In other words, Floquet magnon with opposite spin propagates in opposite directions. This results in a non-vanishing Floquet magnon spin Nernst coefficient with a ${{\mathbb{Z}}}_{2}$ Floquet topological invariant.

## Conclusion

Using a combination of the Floquet theory, spin-wave theory, and quantum field theory, we have presented an exposition of ${{\mathbb{Z}}}_{2}$ magnonic Floquet quantum spin Hall states in irradiated 2D insulating bilayer honeycomb ferromagnets and antiferrmagnets which are coupled antiferromagnetically. These systems have zero net magnetization and preserve ${\mathscr{P}}{\mathscr{T}}$ symmetry. In stark contrast to irradiated graphene^1,5^ and insulating collinear ferromagnets^41,42,50^, we showed that irradiation by circularly-polarized electric field breaks the time-reversal of each honeycomb layer through a photoinduced DM interaction, but ${\mathscr{P}}{\mathscr{T}}$ symmetry of the bilayer antiferromagnetic system is preserved. This results in Floquet spin filtered magnon edge states protected by the ${{\mathbb{Z}}}_{2}$ Floquet topological invariant of the bulk magnon band. The irradiated bilayer antiferromagnetic system exhibits a non-vanishing Floquet magnon spin Nernst effect, whereas the Floquet magnon thermal Hall effect vanishes due to ${\mathscr{P}}{\mathscr{T}}$ symmetry. As we mentioned previously, the bilayer honeycomb chromium iodide CrI_3_^61–63^ is a promising candidate for investigating the current theoretical predictions. We note that topological magnons were recently reported in the ferromagnetic bulk structure of CrI_3_^73^. Therefore, we believe that the current predicted results are pertinent to experiments and will remarkably impact future research in topological insulating antiferromagnets and their potential practical applications to magnon spintronics^31,32^ and photo-magnonics^64^.

## Methods

### Spin wave theory of bilayer honeycomb antiferromagnets

To derive the bosonic Hamiltonian in Eq. (6), we introduce the Holstein Primakoff bosons:

31 $${S}_{i}^{z(\ell )}=S-{a}_{i}^{(\ell )\dagger }{a}_{i}^{(\ell )},{S}_{i}^{+(\ell )}=\sqrt{2S}{a}_{i}^{(\ell )}={({S}_{i}^{-(\ell )})}^{\dagger },$$

for up pointing spins, and

32 $${S}_{i}^{z(\ell )}=-\,S+{a}_{i}^{(\ell )\dagger }{a}_{i}^{(\ell )},{S}_{i}^{+(\ell )}=\sqrt{2S}{a}_{i}^{\dagger (\ell )}={({S}_{i}^{-(\ell )})}^{\dagger },$$

for down pointing spins.

Here ${a}_{i}^{(\ell )\dagger }({a}_{i}^{(\ell )})$ are the bosonic creation (annihilation) operators on the sublattices and ${S}_{j}^{\pm (\ell )}={S}_{j}^{x(\ell )}\pm i{S}_{j}^{y(\ell )}$ denote the spin raising and lowering operators. The bosonic tight-binding models are given by

33 $$\begin{array}{rcl} {\mathcal H}  & = & JS\,\sum _{\langle ij\rangle ,\ell }\,[({a}_{i}^{(\ell )}{a}_{i}^{(\ell )\dagger }+{a}_{j}^{(\ell )}{a}_{j}^{(\ell )\dagger })-({a}_{i}^{(\ell )}{a}_{j}^{(\ell )\dagger }+{\rm{H}}.{\rm{c}}.\,)]\\  &  & +\,\frac{{J}_{c}S}{2}\,\sum _{i,\ell \ne \ell ^{\prime} }\,[({a}_{i}^{(\ell )}{a}_{i}^{(\ell )\dagger }+{a}_{i}^{(\ell ^{\prime} )}{a}_{i}^{(\ell ^{\prime} )\dagger })+({a}_{i}^{(\ell )\dagger }{a}_{i}^{(\ell ^{\prime} )\dagger }+{\rm{H}}.{\rm{c}}.\,)],\end{array}$$

for case (*i*) and

34 $$\begin{array}{rcl} {\mathcal H}  & = & JS\,\sum _{\langle ij\rangle ,\ell }\,[({a}_{i}^{(\ell )}{a}_{i}^{(\ell )\dagger }+{a}_{j}^{(\ell )}{a}_{j}^{(\ell )\dagger })+({a}_{i}^{(\ell )\dagger }{a}_{j}^{(\ell )\dagger }+{\rm{H}}.{\rm{c}}.\,)]\\  &  & +\,\frac{{J}_{c}S}{2}\,\sum _{i,\ell \ne \ell ^{\prime} }\,[({a}_{i}^{(\ell )}{a}_{i}^{(\ell )\dagger }+{a}_{i}^{(\ell ^{\prime} )}{a}_{i}^{(\ell ^{\prime} )\dagger })+({a}_{i}^{(\ell )\dagger }{a}_{i}^{(\ell ^{\prime} )\dagger }+{\rm{H}}.{\rm{c}}.\,)],\end{array}$$

for case (*ii*). The Fourier transform of Eqs (33) and (34) gives the bosonic BdG Hamiltonian in Eq. (6).

### Quantum field theory description of Aharonov-Casher phase

In the presence of an electromagnetic field, the low-energy charge-neutral Dirac magnon near the **K**-point of the BZ is governed by the 2 + 1 dimensional Dirac-Pauli Lagrangian^74,75^

35 $$ {\mathcal L} =\bar{\psi }[-{\varepsilon }_{0}{\gamma }^{0}+{v}_{D}i{\gamma }^{\mu }{\partial }_{\mu }-\frac{{v}_{D}{\mu }_{m}}{2}{\sigma }^{\mu \nu }{F}_{\mu \nu }]\psi ,$$

where ${\varepsilon }_{0}$ accounts for the finite energy Dirac magnon and *v*_*D*_ is the group velocity of the Dirac magnon, and $\bar{\psi }={\psi }^{\dagger }{\gamma }^{0}$. The electromagnetic field tensor is *F*_*μν*_ and ${\sigma }^{\mu \nu }=\frac{i}{2}[{\gamma }^{\mu },{\gamma }^{\nu }]=i{\gamma }^{\mu }{\gamma }^{\nu },(\mu \ne \nu )$ with ${\gamma }^{\mu }=({\gamma }^{0},{\gamma }^{i})$.

To describe the antiferromagnetic Dirac magnon in the presence of an oscillating electric field, we follow the procedure in ref.^76^. In (2 + 1) dimensions, there are two inequivalent representations of the Dirac gamma matrices which generate different Clifford algebras. These two inequivalent representations of the Dirac matrices can be used to describe the ${S}_{z}=1$ and ${S}_{z}=-\,1$ sectors of the antiferromagnetic Dirac magnons. They obey the relation

36 $${\gamma }^{\mu }{\gamma }^{\nu }={g}^{\mu \nu }+i\hat{s}{\epsilon }^{\mu \nu \lambda }{\gamma }_{\lambda }$$

where ${g}^{\mu \nu }={\rm{diag}}(1,-\,1,-\,1)$ is the Minkowski metric and ${\epsilon }^{\mu \nu \lambda }$ is an antisymmetric tensor in (2 + 1) dimensions.

37 $$\hat{s}=i{\gamma }^{0}{\gamma }^{1}{\gamma }^{2}={\gamma }^{0}{\sigma }^{12},$$

with eigenvalues $s=\pm \,1$. We choose the representation

38 $${\gamma }^{0}=(\begin{array}{ll}{\sigma }_{z} & 0\\ 0 & {\sigma }_{z}\end{array}),\,{\gamma }^{1}=(\begin{array}{ll}i{\sigma }_{y} & 0\\ 0 & -i{\sigma }_{y}\end{array}),\,{\gamma }^{2}=(\begin{array}{ll}-i{\sigma }_{x} & 0\\ 0 & -i{\sigma }_{x}\end{array}),$$

such that $\hat{s}$ is diagonal and it is given by

39 $$\hat{s}=(\begin{array}{ll}I & 0\\ 0 & -I\end{array}).$$

In this representation the interaction term transforms as

40 $$\bar{\psi }{\sigma }^{\mu \nu }{F}_{\mu \nu }\psi =-\,\hat{s}{\epsilon }_{\mu \nu \lambda }{F}^{\mu \nu }\bar{\psi }{\gamma }^{\lambda }\psi .$$

The Lagrangian can then be written as

41 $$ {\mathcal L} =\bar{\psi }[\,-\,{\varepsilon }_{0}{\gamma }^{0}+i{v}_{D}{\gamma }^{\mu }{\partial }_{\mu }+\hat{s}{v}_{D}{\mu }_{m}{\gamma }^{\mu }{Q}_{\mu }]\psi ,$$

where ${Q}_{\mu }=(1/2){\epsilon }_{\lambda \nu \mu }{F}^{\lambda \nu }$ is the effective vector potential dual of the field strength tensor. We consider an electromagnetic field with only an oscillating electric field vector $\vec{E}(\tau )$. Hence, ${Q}_{\mu }={\rm{\Xi }}(\tau )=\vec{E}(\tau )\times z$. The Hamiltonian is given by

42 $$H=\int \,{d}^{2}x[\pi (x)\dot{\psi }(x)- {\mathcal L} ]\equiv \int \,{d}^{2}x\,{\psi }^{\dagger }{ {\mathcal H} }_{D}\psi ,$$

where $\pi (x)=\frac{\partial  {\mathcal L} }{\partial \dot{{\rm{\Psi }}}(x)}$ is the generalized momentum. The Hamiltonian is given by

43 $${{\mathscr{H}}}_{D}={v}_{0}+{v}_{D}\vec{\alpha }\cdot (-\,i\vec{{\rm{\nabla }}}-\hat{s}{\mu }_{m}{\rm{\Xi }}(\tau )),$$

where $\vec{\alpha }={\gamma }^{0}\vec{\gamma }$. This is the effective form of the bosonic BdG Hamiltonian in Eq. (6) in the presence of an oscillating electric field vector.

### Floquet-Bloch theory

The time-dependent bosonic BdG Hamiltonian ${\mathscr{H}}(\vec{k},\tau )$ can be studied by the Floquet-Bloch formalism. We can expand it as

44 $${\mathscr{H}}(\vec{k},\tau )=\sum _{n=-{\rm{\infty }}}^{{\rm{\infty }}}\,{e}^{in\omega \tau }{{\mathscr{H}}}_{n}(\vec{k}),$$

where the Fourier components are given by ${{\mathscr{H}}}_{n}(\vec{k})=\frac{1}{T}\,{\int }_{0}^{T}\,{e}^{-in\omega \tau }{\mathscr{H}}(\vec{k},\tau )d\tau ={{\mathscr{H}}}_{-n}^{\dagger }(\vec{k}).$ The corresponding eigenvectors can be written as ${\psi }_{\alpha }(\vec{k},\tau )={e}^{-i{\epsilon }_{\alpha }(\vec{k})\tau }{u}_{\alpha }(\vec{k},\tau )$, where ${u}_{\alpha }(\vec{k},\tau )={u}_{\alpha }(\vec{k},\tau +T)={\sum }_{n}\,{e}^{in\omega \tau }{u}_{\alpha }^{n}(\vec{k})$ is the time-periodic Floquet-Bloch wave function of magnons and ${\epsilon }_{\alpha }(\vec{k})$ are the magnon quasi-energies. We define the Floquet operator as ${{\mathscr{H}}}^{F}(\vec{k},\tau )={\mathscr{H}}(\vec{k},\tau )-i{{\rm{\partial }}}_{\tau }$. The corresponding eigenvalue equation is of the form

45 $$\sum _{m}\,[{{\mathscr{H}}}_{n-m}(\vec{k})+m\omega {\delta }_{n,m}]{u}_{\alpha }^{m}(\vec{k})={\epsilon }_{\alpha }(\vec{k}){u}_{\alpha }^{n}(\vec{k}).$$

Each block Hamiltonian in Eq. (6) obeys this equation.
